# Prescriptions by Clairvoyants

**Published:** 1850-11

**Authors:** 


### Prescriptions by Clairvoyants.

*To the Editor of the New York Medical Gazette.*

Dear Doctor,—The inclosed morceau is at your service. The child referred to in it is about four years old; her disease was simply a mild attack of Infantile Remittent, depending on gastric derangement, and accompanied with a spongy state of the gums, from which a slight hemorrhage occurred. Her parents, previously to sending for me, applied to a

*clairvoyant.* The appended document is the written opinion and prescription. Yours, &c., ALFRED S. PURDY.

NEW YORK, June 1st, 1850.

Copy of a written opinion, by a female somnambulist, after being made clairvoyant by animal magnetism, together with her prescription, the whole *verbatim*, and the original may be seen at this office. We commend it to those M. D.'s who have participated in the spoils of this imposture. In the present case, she was in the pay of a *root* doctor, and she prescribes accordingly. When employed by a homœopathist, she directs the infinitesimal potencies:—

"This child has Scrofulous Tubercles in the Spinal muscles along the neck and back and in the carotid glands. There is a cluster near the lower point of the shoulder-blade that causes pain when excited by cold which darts through the lungs. The liver is tuberculated in clusters of small shot-like lumps, some of which appear to have discharged. The duodenum has a slimy deposit that obstructs some of the most important chyle vessels inducing heated urine at times and bad state of the blood. The diaphragm is weak, as is also the heart and the whole viscera of the chest. The left lung appears to be enlarged in one of its upper lobes and a shriveled state of the lower lobe. There appears to be a deposit of thick matter in the air cells. There are three or four small vessels that have been uncap'd and bled in this part of the lungs into the air tube.

"Dissolve a piece of alum, as large as a walnut, in as little water as will dissolve it, and give ½ teaspoonful every morning.

"Take 2 lbs. raisins, 6 cents' worth slippery elm, 6 cents' worth comfry, 6 cents' Jesuits' bark, spinkenard root 6 cents', 3 cts'. worth gum myrrh, 6 cents' white cohosh—lady slipper root a handful, handful of Green elder Bark, bruise these articles and simmer them in 2 quarts of water, until reduced to 1 quart, then strain and add the liquor in which the raisins have been boiled, then simmer until the whole is reduced to 1 quart, then add 3½ lbs. of sugar and 1 pint of Port wine. Dose ¼ wine glass full three times a day."

---

*To the Editor of the New York Medical Gazette.*

DEAR SIR,—The inclosed written examination and prescription, by a female clairvoyant, is even more absurd and illiterate than the one published in the Gazette of July 6th. The subject of this examination asked the clairvoyant if she was not pregnant? She replied, "No, you are not; you are so much diseased that you could not become so; and if you did, you would not live through it. The result proved the falsity, and exposed the imposition of the clairvoyant, as the patient was safely delivered of a healthy child in about six months after the examination, and has done remarkably well.

The same patient has several written examinations from the same source, but discovering that she was humbugged, has availed herself of rational medicine, and enjoyed better health.

Yours, truly, L. D. MORSE, M. D.,
P. M. South Amboy, New Jersey.

Professional opinion and prescription of a *clairvoyant,* certified by her employer, with his name and residence, in his own chirography and orthography, which is on file:

*New York, Nov. 25th,* 1848.

*Mrs. W.'s Examination*—

Pressure on the Brain, look as if there would be to much water on the brain, if not removed, Bronchial tubes are weak which effects the Eppagladis no disease on the Lungs Great weakness of the Palmonary arteries which arises from the imflamary state of the blood also it has caused a tubucle to form on the Heart, Great weakness in the Heart caused palpatation the blood is in a very bad state which if not removed will cause scrofula disease of the blood, Liver topid and dry muscles swelled, Kidneys are not diseased but are weak caused by the overflowing of the gall on them, Weakness in the womb, Spine is diseased which effects the head and limbs causing a heavy feeling of the whole system.

Rx. Balsam 5 drops every night for one week, Mullien 3 leaves simmered in half Pint Milk drink frequently Cancalagua one teaspoonful three times a day in a little water for one week one gale powder to-night and one Fever powder in the morning. Dr. C. Grattan,

137, *Grand-street, New-York.*

